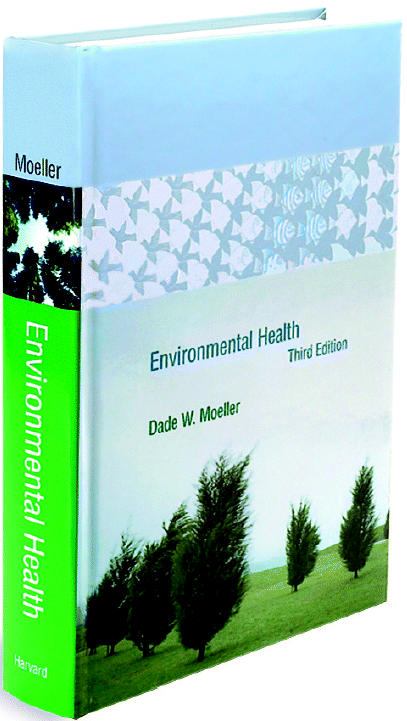# Environmental Health, Third Edition

**Published:** 2005-06

**Authors:** Michael Greenberg

**Affiliations:** Michael Greenberg is professor and associate dean of the faculty at the E.J. Bloustein School of Planning and Public Policy, Rutgers University. His research and teaching focus on environmental health policy; much of it is on clean-up and reuse of contaminated sites in distressed neighborhoods.

By Dade W. Moeller

Cambridge, MA:Harvard University Press, 2005. 606 pp. ISBN: 0-674-01494-4, $65 cloth

At least once a week, I need a brief account of an environmental health area that falls outside my specialty. At those times, I usually am more likely to find what I need in Moeller’s second edition (1997) than in any of my other reference books. This third edition maintains the strengths of the two earlier editions: brief and clear presentations and broad coverage of environmental health.

This edition contains 20 chapters. It begins with an overview and traditional chapters on toxicology and epidemiology. These three are followed by reviews of water, air, and solid waste. About halfway through the 600-page book, the chapters begin to incorporate topics that some environmental health volumes do not cover, such as rodents and insects, food, injury control, environmental economics and law, risk assessment, standards and monitoring, energy, and disasters. A sizeable portion of this material is new or updated for this third edition. For example, you will find new or redone sections on indoor air quality, environmental justice, endangered species, multiple chemical sensitivities, electromagnetic radiation, disasters, and ergonomics. In other words, this book is environmental health in the broadest sense of the word: It is not just pollution control. At the end of each chapter, the author adds an interesting “general outlook” section that summarizes his views of the future.

Two of the most useful chapters are those on electromagnetic radiation and environmental law. The former begins with a description of the electromagnetic spectrum and uses that as a springboard to differentiate among risks associated with different forms of radiation. The chapter briefly reviews highly amplified risks about automobile traffic radar and cell phones and about electromagnetic fields from overhead lines. It also presents useful sections about radon, medical and dental applications, and nuclear materials from electricity-generating stations and nuclear weapons testing. The overall goal is to help readers understand that not all radiation is to be equally dreaded and that some of the most feared radioactive elements are less dangerous than their less feared counterparts. The chapter on environmental law is helpful because the author tries hard to be comprehensive. Moeller offers lists of laws, brief comments on what the laws do and do not accomplish, the trajectory of laws during the last century, and their implementation. This is followed immediately by a chapter on environmental standards that is a good sequel to the law chapter.

Every textbook has limitations. The most obvious is that it takes so long to write one that some of the material is bound to be out of date by the time the book is published. Hence, I would like to have seen more about globalization, pollution prevention, damages to natural resources, terrorism, and life-cycle costs than this edition offers. The author does not ignore these; they are mentioned here and there in the book, but not to the extent needed.

If I have a problem with the book, it is the failure to present a separate chapter on risk perception. I realize that environmental scientists want to operate on the premise that decisions are made using rational thinking processes based on scientific evidence. Unfortunately, as a person who has been studying environmental policy for over 30 years, I say unequivocally that risk perception, trust, and mental models of risk have a major impact on environmental health policies. A chapter needs to be added that includes why people dread some hazards (nuclear weapons, terrorism, handguns) and so their risk is amplified, whereas other hazards are much more tolerable to the public and to elected officials (tobacco, alcohol). Despite this critique, I really like this book, I use it myself, and I will use it in my senior-level undergraduate environmental health course.

## Figures and Tables

**Figure:**